# Pilot of a digital contact tracing card in a hospital setting in New Zealand, 2020

**DOI:** 10.1093/pubmed/fdac045

**Published:** 2022-04-04

**Authors:** Tim Chambers, Andrew Anglemyer

**Affiliations:** Department of Public Health, University of Otago, Wellington 6023, New Zealand; Department of Preventive and Social Medicine, University of Otago, Dunedin 9016, New Zealand

**Keywords:** contact tracing, COVID-19, digital solutions

## Abstract

Countries are rapidly developing digital contact tracing solutions to augment manual contact tracing. There is limited empirical evidence evaluating these tools. We conducted a feasibility study of a Bluetooth-enabled card with hospital staff in New Zealand (*n* = 42). We compared the card data against self-report contact surveys and a stronger Bluetooth device. The cards detected substantially more contacts than self-report contact surveys, while the concordance between Bluetooth devices was high, suggesting that the cards detected clinically relevant close contacts. There was high acceptability among participants, suggesting that their integration would be accepted by healthcare staff. As the pandemic shifts, there is a need to rapidly contact trace and conduct informed risk management, particularly in critical settings such as healthcare.

## Background

Contact tracing, quarantining and monitoring people who were in contact with an infected individual are a key public health response measure in the fight against infectious diseases, including SARS-CoV2 (COVID-19).^1^ Standard contact tracing practice involves constructing self-reported contact histories of cases which are prone to recall and social desirability bias.^2^ Modelling studies suggest that controlling COVID-19 by manual contact tracing alone is challenging,^2^ with outbreaks frequently overwhelming contact tracing capacity.^3^

In response, countries are rapidly developing digital contact tracing (DCT) solutions to augment manual contact tracing systems.^1^ DCT solutions track people’s interactions, creating automated digital contact records between people based on spatio-temporal parameters.^2^ DCT tools have received much attention by researchers to discern their efficacy,^2^^,^^4^^,^^5^ acceptability,^6^ and coherence with privacy laws.^7^ Despite the opportunities of digital proximity tools, there is limited empirical evidence evaluating these tools.^2^ In this short-term feasibility, we aimed to evaluate a proximity tracing tool in a hospital environment, specifically a hardware-based Bluetooth Low-Energy (BLE) DCT card (‘card’ from here on). We aimed to evaluate (i) the card against self-reported close contacts (standard practice), (ii) the card against a stronger Bluetooth device and (iii) assess the acceptability of the card in a hospital setting.

## Methods

### Study design

The study design was a short-term feasibility study. Participants received one card with basic advertising and recording functionality. The card automatically logged interactions with other cards based on hardwired proximity parameters (a close contact algorithm). A full technical assessment of the card was completed by the New Zealand Defence Technology Agency.^8^ Additionally, a closed field trial of the card was completed where everyday scenarios were conducted, recorded and tested for accuracy.^9^ In short, the cards recalled greater than 90% of close contacts, with a 36% false positive rate (at the threshold of 15 minutes within 2 m).

To compare the cards against standard contact tracing practice, all participants self-reported close contacts in a daily contact diary online. In a secondary analysis, a subset of participants (*n* = 5), which changed daily, also received an ultra-wide band (UWB) Bluetooth device (see Supplementary Fig. 1, See online supplementary material for a colour version of this figure), data from which we compared to card data. The UWB Bluetooth device was a physical distancing system (https://www.bump-space.com/) produced by Tharsus, UK. It contains a rechargeable lithium-ion battery and uses IEEE 802.15.4 UWB wireless technology to perform time-of-flight ranging with centimetre-level accuracy.

The study took place within a medical unit at a hospital in New Zealand over a 6-day period from 7 May to 13 May 2020, allowing for both weekday and weekend interactions. The study population for the trial included healthcare workers from a single medical unit at a hospital. We enrolled all interested participants until we distributed all hardware (~60% of eligible staff). Previous analyses have shown that at least 56% of people would be required for Bluetooth-enabled apps to be effective in contact tracing.^5^

### Exit survey

At the conclusion of the pilot, we distributed a brief, anonymous cross-sectional survey soliciting information about willingness to document personal contacts using various methods and general feedback on the pilot.

### Data analysis

The BLE card data, UWB data, daily self-report close contact questionnaires and qualitative data from exit interviews were summarized. To determine the efficacy of the card, we assessed the concordance between close contact data by summarizing the total number of contacts and duration of time per contact for BLE cards and self-report surveys and qualitatively compared them (see Supplementary Table 1). In a secondary analysis, we compared card data with data from UWB devices.

## Results

In total, there were 42 participants included in the study (Table 1). This represented ~60% of the eligible staff population but also represented staff with the most staff-to-staff interactions (e.g. more nurses than senior clinicians with their own office). The number of participants who were actively working each day during the feasibility study ranged from 13 to 35 across all 6 days. There was a technical problem with the firmware on the BLE card that led to all data before 12 pm on Day 2 being discarded—reducing the observation period to 5 days.

**Table 1:** Study participant demographics (*n* = 42)

Demographic characteristic		n (%)
Total	*Total*	42 (100%)
Ethnicity	*NZ European*	28 (66.7%)
	*Asian*	7 (16.7%)
	*Other European*	4 (9.5%)
	*NZ European/Māori*	3 (7.1%)
Role	*Nurse*	18 (42.9%)
	*Junior Doctor*	9 (21.4%)
	*Allied Health Team Member*	5 (11.9%)
	*Senior Doctor*	4 (9.5%)
	*Ward Administrative Staff*	3 (7.1%)
	*Ward Domestic Staff*	2 (4.8%)
	*Other (CNM)*	1 (2.4%)

### Concordance between Bluetooth cards and self-report daily diaries

**Fig. 1:**
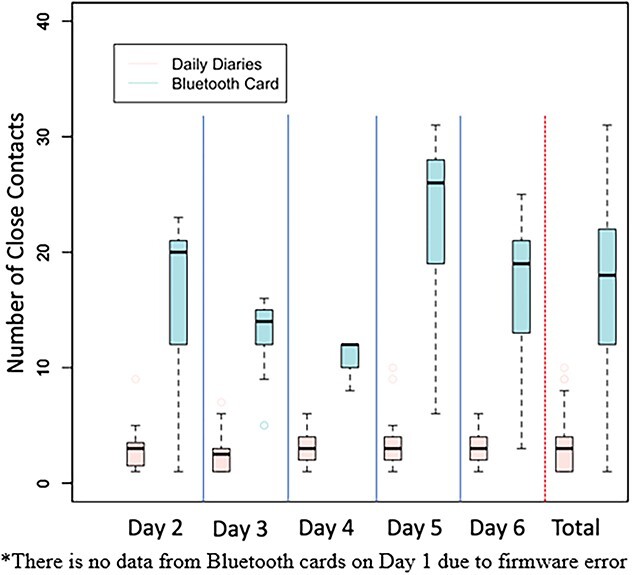
Comparison of close contacts detected by Bluetooth card and self-report surveys.

The median number of close contacts recorded per day over the study period was 18 for the cards compared with only 3 for the self-report surveys (see Fig. 1). The median duration of time spent with a close contact was 30 minutes for the self-report surveys compared with 46 minutes for the card (see Supplementary Table 1).

### Concordance between cards and UWB devices

There were 78 UWB entries in the data from 5 days. Fifty (64%) of these entries matched card records. Of the 28 unmatched records, 16 (57%) were from the 7th of May for where there was no valid card data due to a technical fault in the firmware. The remaining 12 (43%) unmatched entries (from 4 different participants) were from participants who had no Bluetooth card data on those days. This was a result of discrepancies between the work roster provided to the researchers and the true work roster. Thus, the card-UWB concordance was high, albeit without regard for potential false positives.

### Acceptability of cards by healthcare staff

The response rate for the exit survey among participants was less than anticipated (38%). All study participants were willing to use a DCT card again (see Supplementary Table 2). When compared with other potential DCT solutions, the card was the most acceptable, followed by a smartphone app that automatically recorded contacts.

## Conclusions

The cards provided greater recall of close contacts than self-report surveys, which suggests that DCT tools are more effective at detecting close contacts than standard contact tracing practice. The high concordance between cards and UWB devices suggests that the cards could consistently detect true positives. The ability to detect true positives is also supported by the median duration of time spent between contacts recorded by the cards (e.g. >30 minutes), which suggest that these are unlikely to be false positives. A separate trial concluded that the card had a false-negative rate of 10% and false positive rate of 36% (most would still be defined as casual contacts).^9^

Participants showed a high degree of acceptability of DCT in the workplace settings. Firstly, the majority (~60%) of the ward asked to participate on very short notice, particularly those in more high-risk roles (e.g. nurses). Secondly, the interview data underscored the willingness of participants to be involved. The observed acceptability levels are much higher than surveys in the general public, though our response rate from participants was low.^6^

The reliance on contact tracing has waned in countries that have adopted a mitigation COVID-19 response strategy. In contrast, contact tracing has remained a core element of the COVID-19 response for those countries adopting elimination or aggressive suppression strategies like New Zealand. However, even New Zealand has recently indicated a shift to a mitigation strategy which de-emphasized the role of manual contact tracing.^10^ In this environment, DCT solutions function to notify citizens of their potential exposure so they can make informed decisions around contact with vulnerable people close to them or used by Governments to prioritize the limited testing resources.

As the pandemic shifts, there may be a stronger use case for DCT in critical settings such as healthcare. For example, in New South Wales Australia, an estimated 2000 healthcare staff were ordered to self-isolate as identified cases or close contacts placing enormous pressure on the system.^11^ DCT solutions provide a more accurate way of determining a close contact, with limited human intervention, which can also be adapted in response to the current system demands or definition of close contacts. This could involve shifting spatio-temporal thresholds to require longer exposure events to classify a close contact (spatio-temporal risk management) or prioritize isolation for those close contacts that encountered multiple cases (cluster-based risk management).

Among the limitations in this real-world application of DCT card is the lack of close surveillance of card interactions. However, a previous analysis assessed the false positive and false negative rates of the card.^9^ Second, this feasibility study suffered from a number of technological issues.

DCT technologies are rapidly being deployed to help combat the COVID-19 pandemic. Our feasibility study of a Bluetooth-enabled card provides some additional empirical evidence of their potential utility in one setting. Future research needs to be focused on the assessment of DCT performance in real-world settings and determine the acceptability of these solutions in those settings.

## Supplementary Material

supplementary_fdac045

## Notes

**Tim Chambers,** Senior Research Fellow

**Andrew Anglemyer,** Senior Research Fellow
