# Supplementary material for: Pilot of a digital contact tracing card in a hospital setting in New Zealand, 2020
Source: J Public Health (Oxf). 2022 Apr 4;45(2):e171–4. doi: 10.1093/pubmed/fdac045 (PMC9383627; doi:10.1093/pubmed/fdac045)
Supplement: supplementary_fdac045 [file supplementary_fdac045.docx]

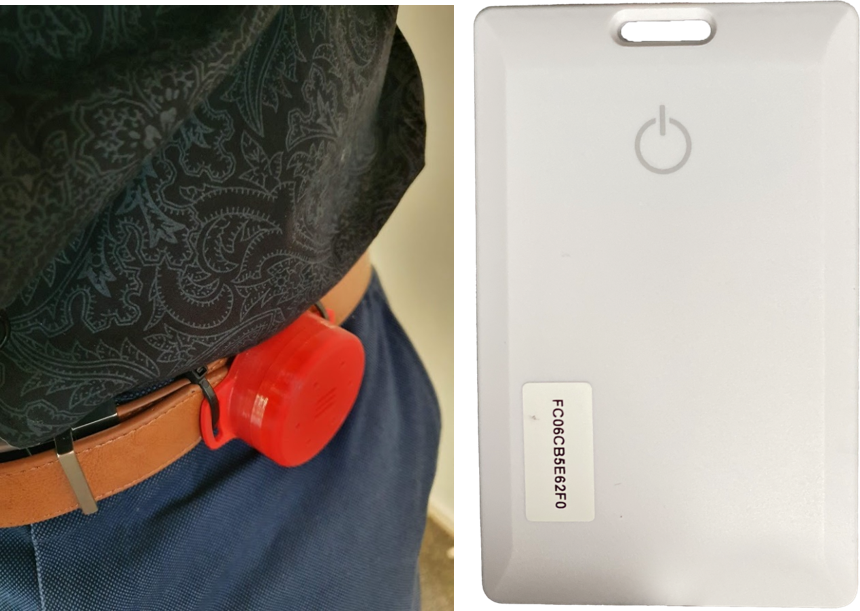


Supplementary Figure 1. The ultra-wide band Bluetooth device (left) and prototype Bluetooth card (right)

Supplementary Table 1. Duration (in minutes) per close contact, as reported in daily diaries and Bluetooth cards

| Day | Median duration per self-reported survey close contact in minutes by day (range) | Median duration per Bluetooth card close contact in minutes by day (range) |
| --- | --- | --- |
| Day 1: Thursday*  Day 2: Friday  Day 3: Saturday  Day 4: Sunday  Day 5: Monday  Day 6: Tuesday  Total | 20 (10-420)  25 (10-480)  25 (10-300)  15 (10-180)  40 (10-240)  30 (10-300)  30 (10-480) | No Data  38 (10-241)  60 (10- 317)  40 (10-216)  46 (10-312)  53 (10-250)  46 (10-317) |

*There is no data from Bluetooth cards on Day 1 due to firmware error

Supplementary Table 2: Willingness to document close contacts using various methods

|  | *Not at all willing*  *n* (%) | *Somewhat willing*  *n* (%) | *Very willing*  *n* (%) | *Missing*  *n* (%) |
| --- | --- | --- | --- | --- |
| Method  Personal diary on paper  Personal diary on-line  Entering data yourself in book  Entering data yourself in computer  Use a passive app on smart phone  Use an active app on smart phone  Use a contact card (i.e., Bluetooth card) | 10 (62.5)  8 (50.0)  5 (31.3)  3 (18.8)  3 (18.8)  3 (18.8)  0 (0.0) | 4 (25.0)  6 (37.5)  9 (56.3)  11 (68.8)  2 (12.5)  8 (50.0)  2 (12.5) | 1 (6.3)  1 (6.3)  1 (6.3)  1 (6.3)  11 (68.8)  5 (31.3)  13 (81.3) | 1 (6.3)  1 (6.3)  1 (6.3)  1 (6.3)  0 (0.0)  0 (0.0)  1 (6.3) |

**Exit survey questions**

*Preamble*

*Records need to be kept in case people need to be traced if there are new cases of COVID-19. This can be a diary or guest book at places we visit. Or we could use apps placed on our mobile phones or contact tracing cards that use Bluetooth technology. Here is some information about apps and Bluetooth. Please read this information before we ask you three questions:*

*Bluetooth technology registers when you are near to other Bluetooth devices eg. when you meet someone face to face / ā kānohi, or when you stand near to someone in the supermarket queue. Bluetooth does not record any data on your location (GPS) and does not track your movements.*

*Passive apps are downloaded onto your smartphone and automatically record when you are near to other Bluetooth devices.*

*Active apps would also be downloaded to your smartphone but would require you to scan a barcode as you entered a store (for example) and to manually enter people you been near to that day.*

*Contact tracing cards using Bluetooth like the ones used in this study that automatically record when you are near to cards carried by other people.*

*We would like to know what you think of options to record when you are near to other people in public places -*

How are you currently recording details of when you are near to other people? Tick one

I keep a full written record of everyone I am near to in public places

I keep a written record of most of the people I am near to in public places

I write my details into the guest book of a store or venue

I do not keep a written record of people I am near to in public places

I have a contact app installed on my smartphone (please specify app name) ________________

If you had to record details of people you have been near to in public places, which of the following would you be willing to use? (not at all willing/ quite willing/ very willing)

Personal daily diary (paper)

Personal daily diary (online)

Writing my personal contact details in a book provided at each store or venue

Entering my personal contact details into a computer at each store or venue

Passive app on my smartphone that automatically records Bluetooth devices near me

Active app on my smartphone that I use to sign into stores and manually record the details

Contact card that automatically records other cards carried by people near me.

Which of those options would you prefer to use if you had to record details of people you have been near to in public places? tick one only

Personal daily diary (paper)

Personal daily diary (online)

Writing my personal contact details in a book provided at each store or venue

Entering my personal contact details into a computer at each store or venue

Passive app on my smartphone that automatically records Bluetooth devices near me

Active app on my smartphone that I use to sign into stores and manually record the details

Contact card that automatically records other cards carried by people near me.

Other (please specify) __________________________
